# Application of photoacoustic computed tomography in biomedical imaging: A literature review

**DOI:** 10.1002/btm2.10419

**Published:** 2022-09-29

**Authors:** Yanru Gu, Yuanyuan Sun, Xiao Wang, Hongyu Li, Jianfeng Qiu, Weizhao Lu

**Affiliations:** ^1^ Department of Radiology The Second Affiliated Hospital of Shandong First Medical University Taian China; ^2^ Department of Radiology Shandong First Medical University and Shandong Academy of Medical Sciences Taian China; ^3^ College of Ocean Science and Engineering Shandong University of Science and Technology Qingdao China

**Keywords:** animals, biomedical imaging, humans, photoacoustic computed tomography

## Abstract

Photoacoustic computed tomography (PACT) is a hybrid imaging modality that combines optical excitation and acoustic detection techniques. It obtains high‐resolution deep‐tissue images based on the deep penetration of light, the anisotropy of light absorption in objects, and the photoacoustic effect. Hence, PACT shows great potential in biomedical sample imaging. Recently, due to its advantages of high sensitivity to optical absorption and wide scalability of spatial resolution with the desired imaging depth, PACT has received increasing attention in preclinical and clinical practice. To date, there has been a proliferation of PACT systems designed for specific biomedical imaging applications, from small animals to human organs, from ex vivo to in vivo real‐time imaging, and from simple structural imaging to functional and molecular imaging with external contrast agents. Therefore, it is of great importance to summarize the previous applications of PACT systems in biomedical imaging and clinical practice. In this review, we searched for studies related to PACT imaging of biomedical tissues and samples over the past two decades; divided the studies into two categories, PACT imaging of preclinical animals and PACT imaging of human organs and body parts; and discussed the significance of the studies. Finally, we pointed out the future directions of PACT in biomedical applications. With the development of exogenous contrast agents and advances of imaging technique, in the future, PACT will enable biomedical imaging from organs to whole bodies, from superficial vasculature to internal organs, from anatomy to functions, and will play an increasingly important role in biomedical research and clinical practice.

## 1 INTRODUCTION

Photoacoustic computed tomography (PACT) is a major manifestation of photoacoustic tomography (PAT).^1^ PACT is based on the photoacoustic effect, where biological tissues absorb the incident light, and generates acoustic waves in ultrasound range, the acoustic waves are detected by traditional ultrasound transducers, and cross‐sectional and 3D images are finally reconstructed from the acoustic waves.^1^ Recently, there has been increasing interest surrounding the use of PACT in biomedicine. PACT, as a hybrid imaging modality, achieves light absorption measurement and acoustic detection simultaneously.^2^, ^3^, ^4^ While the resolution and imaging depth of optical imaging modalities such as confocal microscopy, two‐photon microscopy, and optical‐coherence tomography are limited by the diffraction barrier and optical diffusion limit, respectively,^1^, ^5^ PACT overcomes the diffraction and diffusion limits of these imaging modalities to enable imaging with deep penetration, high spatiotemporal resolution, and rich contrast.^1^, ^2^, ^3^, ^4^

Currently, the biomedical applications of PACT include both preclinical research and clinical applications.^1^ Preclinical research focuses on small animals, such as brain imaging, whole‐body vascular density imaging, and whole‐body functional imaging of mice.^6^, ^7^ Jose et al. performed hybrid imaging of light absorption, speed of sound, and acoustic attenuation in 2010.^8^ Xia et al. reported a novel small‐animal whole‐body imaging system called ring‐shaped confocal PACT (RC‐PACT) in 2012, which provides a series of cross‐sectional images of the mouse brain, liver, kidney, and bladder.^7^ Using the RC‐PACT system, Chatni et al. demonstrated that both anatomy and glucose uptake can be imaged with a single modality.^9^ Yao et al. demonstrated the feasibility of imaging mouse brain metabolism using PACT in 2012.^10^ Meng et al. developed ultrasonic‐array‐based optical‐resolution PACT in 2012.^11^ Xia et al. introduced retrospective respiratory gating for whole‐body PACT in 2014.^12^ In 2016, Li et al. demonstrated that PACT can provide high‐resolution label‐free imaging of structures in the entire mouse brain.^13^ Mitsuhashi et al. first proposed transcranial PACT brain imaging in 2017.^14^ In the same year, Li et al. imaged in vivo whole‐body dynamics of small animals by using single‐impulse panoramic PACT and achieved high spatiotemporal resolution.^6^ In 2018, Wang et al. found that ingestible baked barley can enhance the contrast of photoacoustic imaging.^15^ Later, Li et al. imaged both a 7.5‐cm deep leaf target embedded in an optically scattering medium and the beating heart of a mouse overlaid with 3.7‐cm thick chicken tissue via internal light illumination combined with external ultrasound detection to improve the penetration depth of PACT.^16^ In 2019, Liang et al. investigated the impact of the murine skull on the noninvasive PACT, which paved the way for future PACT of mouse brains.^17^ Lv et al. achieved structural and functional imaging of ischemic stroke via a bowl‐shaped array PACT in 2020.^18^ Later in the same year, Li et al. introduced an ergodic relay into the PACT system, which had the potential for functional imaging and biometric application in vivo.^19^ In 2021, Huang et al. used multispectral optoacoustic tomography (MSOT) to visualize lipids in laboratory mice, and demonstrated that MSOT was an efficient imaging technique for lipid visualization in the preclinical model of fatty liver disease.^20^ In 2022, Vagenknecht et al. demonstrated tau protein imaging in mice brain using tau‐targeted pyridinyl‐butadienyl‐benzothiazole derivative PBB5 and MSOT.^21^

Clinical research of PACT mainly focuses on the examination of human breast cancer and skin melanoma.^22^, ^23^ Wang et al. demonstrated that compact lasers based on emerging diode technologies are well suited for preclinical and clinical PACT.^24^ Jose et al. studied the applicability of PACT imaging as an intraoperative modality for examining the status of resected human sentinel lymph nodes in 2011.^23^ Es et al. investigated the use of PACT to visualize the blood vessels in a healthy human finger, focusing on vascularity across both interphalangeal joints, in 2014.^25^ Biswas et al. created a method for delineating surfaces of the finger's bone, which can be used to detect rheumatoid arthritis, in 2015.^26^ Meng et al. developed a principal component analysis‐based PACT in 2016 and used it to obtain in vivo images of the vasculature of a human hand.^27^ Also in 2016, Liu et al. developed and validated a dual‐modality imaging technique, namely, MPAUCT, which combines multispectral PACT and ultrasound computed tomography for imaging of human finger joints.^28^ Lin et al. achieved PACT imaging of human breast tissue by scanning the entire breast within a single breath‐hold (SBH; 15 s) in 2018, which was the first time PACT had been used for human breast cancer detection. They imaged the breasts of seven breast cancer patients and identified seven of eight tumors successfully.^22^ Via a self‐developed PACT system, Wray et al. obtained volumetric image of angiographic structures in human extremities including arms and legs in 2019.^29^ Later that year, Yang et al. used a co‐registered PACT and ultrasound system to image ex vivo human colorectal tissue samples, and showed the potential of PACT in the diagnosis of colorectal cancer.^30^ In 2020, Yang et al. developed a concave transducer array‐based PACT system, and realized hemodynamic monitoring of the foot vessels in diabetic patients via the PACT system.^31^ Shan et al. demonstrated the feasibility of PACT in monitoring fetal vascular dynamics in the same year.^32^ In 2021, Liapis et al. used an eigenspectral MSOT to evaluate chemotherapeutic effects on breast tumor hemodynamics.^33^ Via massively parallel transducers‐based PACT system, Na et al. achieved human brain functional imaging.^34^

PACT has emerged as a novel imaging modality that provides high‐resolution imaging of optical absorption in deep tissue. Over the past few years, various preclinical and clinical applications of the technique have been demonstrated, including functional brain imaging, small‐animal whole‐body imaging, breast cancer screening, and guidance of lymph node biopsy.^1^ In this review, we searched the Web of Science database for articles published during the past two decades using keywords including PACT, biomedical, animal, and human. And the article reviews the applications of PACT in biomedical research and clinical practice with the following structure: Section 2 introduces PACT, contrast agents and reconstruction algorithms. Section 3 describes the preclinical studies using PACT. Section 4 summarizes the clinical applications of PACT. Finally, Section 5 summarizes the status of PACT in biomedicine and discusses the future directions of PACT.

## 2 PACT SYSTEMS IN BIOMEDICAL IMAGING

### 2.1 A brief introduction to PACT

PACT uses nonionizing laser illumination to generate a local temperature rise in the biological tissues, which is subsequently converted to transient thermoelastic expansion and ultrasonic emission. The generated ultrasonic waves are detected by acoustic transducers, and the temporal signals are reconstructed to form an image of the optical absorbers.^4^ A PACT system generally consists of laser, light path system, imaging plane, acoustic detector, and post‐processing platform.

An optical parametric oscillator pumping laser is typically used as the light source in PACT systems because it can deliver nonionizing laser pulses to the object and lead to thermal expansion. A light path system is designed for diffracting or focusing a beam of light, and it may include a linear microlens array or a diversified objective lens depending on the imaging goal. The light beams are focused on the imaging platform where the target imaging object is placed. Then, the acoustic transducer measures conventional acoustic signals from the object. Finally, the image is reconstructed from the received PA signals via various algorithms in the post‐processing platform.^8^

### 2.2 PACT systems applied in biomedical imaging

**FIGURE 1:**
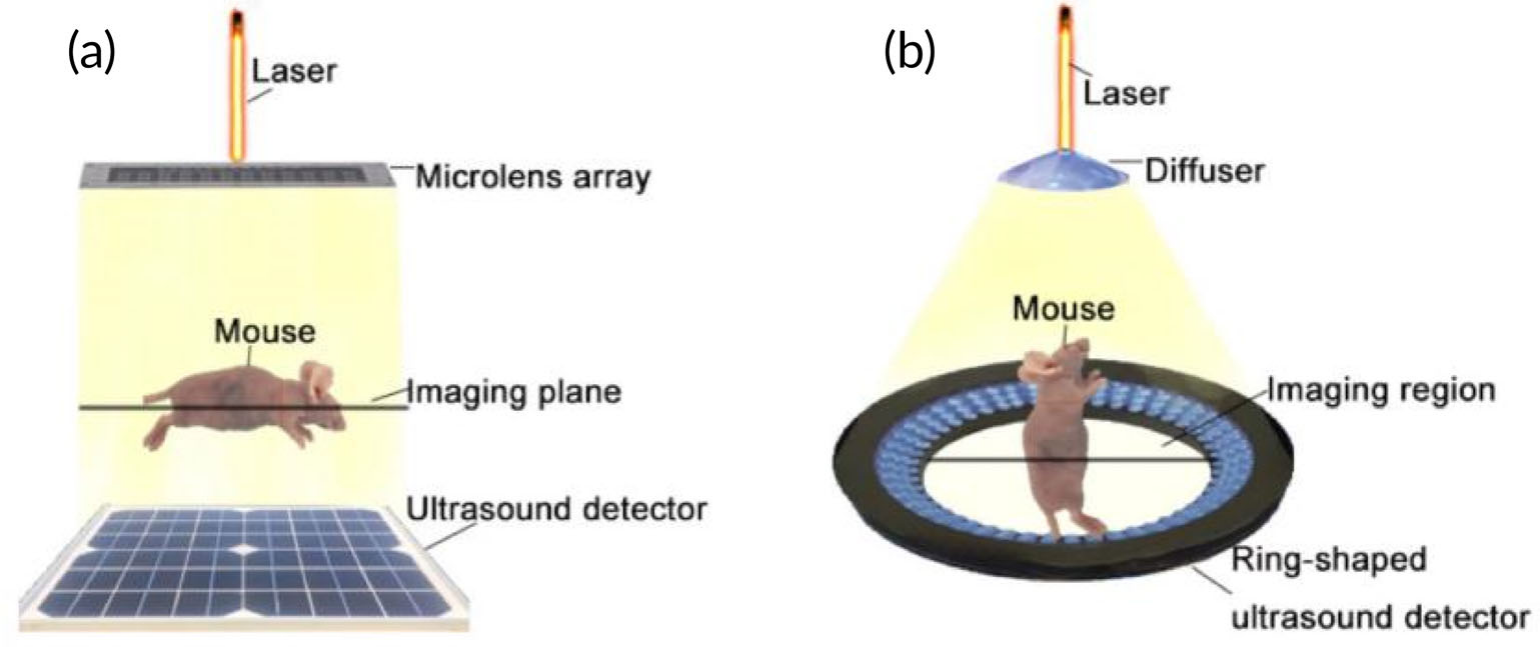
Two types of photoacoustic computed tomography (PACT) systems. (a) The first type of PACT system with incident light perpendicular to the signal receiving surface of the transducer. (b) The second type of PACT system with incident light parallel to the signal receiving surface of the transducer

UP to now, several PACT systems with different configurations have been proposed and used in biomedical imaging. According to the perpendicular or parallel relationship between incident light and the signal receiving surface of the detector, PACT systems can be roughly classified into two types^6^, ^7^, ^8^, ^11^, ^22^, ^35^ (Figure 1). The transducer of the former (Type I) is usually rectangular or arc‐shaped, volumetric images are obtained by rotating the transducers or rotating the imaging object^8^, ^11^, ^20^ (Figure 1a). The transducer of the latter (Type II) is usually ring‐shaped to obtain cross‐sectional images.^6^, ^7^, ^22^, ^35^ Three‐dimensional images are obtained by scanning the imaging object elevationally^6^, ^7^, ^22^, ^35^ (Figure 1b).

In terms of the number of acoustic transducers, Type I PACT usually contains dozens of elements in the transducer array,^8^, ^11^, ^20^ Type II PACT contains several hundreds of elements in general.^6^, ^7^, ^22^, ^35^ However, there were other PACT configurations which contains few elements in the acoustic transducer, for instance, the PACT platform configured by Li et al. only used one‐element acoustic transducer, which detects widefield PA signals encoded by the ergodic relay.^19^ Table 1 summaries the representative PACT systems applied in biomedical imaging.

**TABLE 1:** PACT systems in biomedical imaging

PACT systems	Imaging object	Introduction	Type	Reference
PER‐PACT	Specially designed phantoms and biological specimens (muscle and adipose tissue)	PER‐PACT can simultaneously image both optical absorption properties and acoustic transmission properties of an object in two‐dimensional slices by adding a “passive” element to a computed tomography photoacoustic imager.	Type I	^8^, ^36^
OR‐PACT	The ear of an anesthetized nude mouse	OR‐PACT is developed by mechanically scanning a focused laser beam together with a confocal ultrasonic transducer, for achieving micrometer or even submicrometer lateral resolution with faster imaging speed.	Type I	^11^
RC‐PACT	Whole‐body of the mouse	RC‐PACT is based on a confocal design of free‐space ring‐shaped light illumination and 512‐element full‐ring ultrasonic array signal detection. It enables fast and accurate tomographic inversion of full‐view cross‐sectional images.	Type II	^7^, ^9^
Multi‐angle illumination PACT	Blood vessel phantoms, ex vivo sheep head	Multi‐angle illumination PACT is based on the multi‐angle illumination technique and a linear‐array transducer. Via multiple incident light paths, the interaction time between the incident photons and biological tissues are longer, leading to improved image quality and greater penetration depth.	Type I	^37^
RS‐PACT	Mouse brain, ear and liver.	RS‐PACT is upgraded from RC‐PACT, with two laser sources, which can switch on and off the protein‐based phytochromes, and a full‐ring acoustic transducer array. RS‐PACT enables monitoring tumor growth and metastasis with 100 μm resolution at the 10 mm depth.	Type II	^38^
SIP‐PACT	Functional images of brain and whole‐body of the mouse, ex vivo breast tumor	SIP‐PACT employs a 512‐element full‐ring acoustic transducer array with simultaneous one‐to‐one mapped preamplification and analogue‐to‐digital sampling. It simultaneously integrates high spatiotemporal resolution, deep penetration, multiple contrasts, full‐view fidelity, and high detection sensitivity.	Type II	^6^, ^39^
SBH‐PACT	Human breasts	The SBH‐PACT system mainly comprises of an illumination laser, an ultrasonic transducer array, signal amplification/acquisition modules, a linear scanning stage, and a patient bed. It integrates deep penetration, high spatiotemporal resolution, sensitive breast cancer detection, and 2D/3D switchable modes.	Type II	^22^, ^40^
DS‐PACT	Biological tissues (bovine tendon) buried deep inside scattering media	DS‐PACT can boost dichroic signals from biological tissues by modulating the polarization of linearly polarized light and measuring the alternating signals through lock‐in detection. It can image the dichroism of biological tissues and detect their orientations at a depth much beyond the ballistic regime.	Type II	^35^
MSOT	Breast cancer, vascular, skin and lymph nodes imaging of human, functional imaging of lipids in the mice liver	MSOT collects images at multiple wavelengths and resolves the spectral signatures in each voxel in the images, making it a multispectral method. The MSOT mainly consists of a tunable laser source which guarantees acquiring images at multiple wavelengths, and a cylindrically focused acoustic transducer array or a handheld probe. MSOT enables anatomic, functional and molecular imaging simultaneously.	Type I or Type II	^20^, ^21^, ^28^, ^33^
PATER	Mouse brain, vascular imaging	PATER images widefield PA signals encoded by the acoustic ergodic relay with a single‐laser shot. PA waves are encoded inside the ergodic relay and finally detected by a single‐element transducer.	Type I	^19^, ^41^
Internal‐illumination PACT	Rats deep brain, a mouse overlaid with 37 mm chicken breast tissue	Internal‐illumination PACT uses a multimode optical fiber as the light delivery scheme to the deep structures inside the living rats and mice. Either full‐ring shaped transducer array or linear shaped transducer array can be used to receive the PA signals.	Type I or Type II	^16^, ^42^, ^43^

Abbreviations: DS, dichroism‐sensitive; MSOT, multispectral optoacoustic tomography; OR, optical‐resolution; PACT, photoacoustic computed tomography; PATER, photoacoustic topography through an ergodic relay; PER, passive element enriched; RC, ring‐shaped confocal; RS, reversibly switchable; SBH, single‐breath‐hold; SIP, single‐impulse panoramic.

### 2.3 Endogenous and exogenous contrast agents

Since the amplitude of the PA signal is sensitive to optical absorption, variations of biological tissues in optical absorption introduce imaging contrast in PACT imaging.^44^, ^45^ In biomedical PACT imaging, materials that absorb optical signal can be endogenous or exogenous, and are known as endogenous or exogenous contrast agents.^45^

#### 2.3.1 Endogenous contrast agents

Endogenous contrast agents are biomolecules inside the body; therefore, the main advantage of imaging them with PACT is the label‐free capability with high optical absorption contrast.^45^ Endogenous chromophores mainly include hemoglobin, melanin, deoxyribonucleic acid (DNA), ribonucleic acid (RNA), lipids, and water.^45^

Hemoglobin is the dominant absorber of light in the visible and near‐infrared (NIR) part of the optical spectrum and is commonly used for PACT imaging.^46^ Imaging hemoglobin with PACT can not only visualize vascular anatomy,^4^ but also provide functional information associated with blood oxygen saturation (sO_2_).^47^, ^48^, ^49^ Melanin is another important endogenous absorber, which is sensitive to visible and NIR light around 750 nm.^4^, ^22^, ^23^ Since melanin is produced by melanocytes, it can be used to reveal melanomas and metastatic melanoma cells.^50^ DNA and RNA in cell nucleus have strong optical absorption at 200–300 nm deep ultraviolet wavelength region.^1^ To date, via the DNA/RNA endogenous contrast, researchers have imaged cell nuclei using photoacoustic microscopy.^51^ Lipids are implicated in various diseases; therefore, lipids visualization gains increasing attention.^20^ Lipids have strong optical absorptions around NIR wavelengths, with the absorption peak occurring at 930 nm.^45^ Imaging lipids with PACT has enabled the visualization of hepatic steatosis in a preclinical mice model,^20^ breast tumors,^52^ and arterial walls.^53^ Water absorbs strongly at NIR wavelengths longer than 900 nm, with a strong peak at 980 nm.^45^ Generally, different endogenous contrast agents have different optical absorption properties, as long as the corresponding wavelength range is used to illuminate the samples, PACT can detect the corresponding endogenous absorbers.

#### 2.3.2 Exogenous contrast agents

Although endogenous contrasts have played critical roles in biomedical PACT imaging, they can only visualize limited biological processes because not all tissues have distinct optical absorption properties.^45^ To overcome this issue, target‐specific exogenous contrast agents for PACT have been developed by researchers.^54^

The most common type of exogenous contrast agents is nanoparticle.^55^ To date, various nanoparticles have been designed for PACT imaging.^55^, ^56^ Metallic nanoparticles, especially gold nanoparticles including spheres, rods, shells, and cages are commonly used contrast enhancers in PACT for imaging specific biological processes, such as inflammatory response,^57^, ^58^ tumor,^54^ macrophages,^59^ and melanoma.^60^ In addition to gold, other metallic nanoparticles, such as silver, platinum, copper, and palladium have been used to generate contrast in PACT imaging.^45^, ^55^, ^61^ Carbon nanotubes are another class of nanoparticles that have been used as contrast enhancers to improve contrast and sensitivity of tumor and angiogenesis imaging in mice.^62^, ^63^

Although nanoparticles are commonly used in PACT, concerns remain as they may lead to potential cytotoxicity and inflammatory response.^45^ Organic contrast agents overcome the disadvantages of the metallic nanoparticles and carbon nanotubes, as they are nontoxic and biocompatible.^54^, ^64^, ^65^ Some organic dyes absorb NIR light and can lead to enhanced contrast in PACT imaging.^54^ For instance, via indocyanine green‐based contrast enhancement, target‐specific PACT tumor imaging has been realized.^66^, ^67^ Methylene blue has been assisted to visualize lymph nodes in humans and rats.^64^, ^68^, ^69^ Other dye‐based exogenous contrast agents such as Evans blue, phthalocyanine have been used in PACT imaging.^44^ Organic contrast agents such as semiconducting polymer, semiconducting small molecules have also been used to fabricate NIR‐I and NIR‐II PACT contrast agents.^55^, ^64^, ^65^, ^70^, ^71^ In addition, via some organic contrast agents, protein imaging can be achieved. For instance, tau‐targeted pyridinyl‐butadienyl‐benzothiazole derivative PBB5 can aid tau‐protein imaging in the mice brain.^21^ There are also protein‐based phytochromes, such as RpBphP1, RpBphP3, and DrBphP phytochromes, which are binded with some heme‐derived biliverdin chromophore and can aid the detection of protein–protein interactions in tumors and livers of mouse models via RS‐PACT and SIP‐PACT configurations.^38^, ^72^

### 2.4 PACT imaging reconstruction algorithm

In a PACT system, acoustic transducers are used to collect the PA signals from biological tissues, and then reconstruction algorithms are applied to reconstruct light absorption images.^73^ Currently, reconstruction methods for PACT can be divided into two main categories: conventional reconstruction methods and learning‐based reconstruction methods.^74^

Conventional reconstruction methods include direct reconstruction (back‐projection algorithms and filtered back‐projection algorithms)^75^, ^76^ and model‐based iterative reconstruction.^77^, ^78^ Direct reconstruction mainly involves solving a single wave equation to represent the idealized mathematical model of the PACT system and to recover the image from the tissue of interest.^79^ Therefore, direct reconstructions are of high computational efficiency.^74^ However, PACT images reconstructed by direction reconstructions will degrade under the condition of sparse sampling.^73^ In addition, direction reconstruction methods usually ignored acoustic heterogeneity and used a constant speed of sound for image reconstruction.^80^ Model‐based iterative reconstruction aims to reconstruct the PACT images by solving the optimization problem iteratively.^74^ This reconstruction technique can address the inhomogeneous acoustic properties of the imaging object,^77^ such as dual‐speed reconstruction,^6^ joint reconstruction,^81^ and ultrasound computed tomography enhanced reconstruction.^82^ Model‐based iterative reconstruction methods usually assume multiple speed of sound during image reconstruction, which efficiently address the acoustic heterogeneity.^80^ However, model‐based iterative reconstruction is computationally intensive.^73^, ^74^, ^83^

Learning‐based reconstruction methods for PACT are based on machine learning algorithms such as directory learning and deep learning algorithms.^73^, ^74^, ^83^ Learning‐based reconstruction methods can learn end‐to‐end transformation from both direction reconstructions and model‐based iterative reconstructions, and can further reduce the artifacts and noise generated from the conventional reconstruction methods.^74^ To date, several deep learning algorithms including U‐Net and its variants,^77^, ^83^ Y‐Net and its variants,^74^, ^84^ have been developed for PACT image reconstruction with enhanced image quality and spatial resolution.^80^ Recently, generative adversarial network (GAN) and its variants including Wasserstein GAN, knowledge infusion GAN,^74^, ^83^, ^85^ have been used to approximate the real PA data, and have achieved superior performances in terms of signal‐to‐noise ratio and structural similarity.^83^

## 3 THE APPLICATION OF PACT IN ANIMAL IMAGING

**FIGURE 2:**
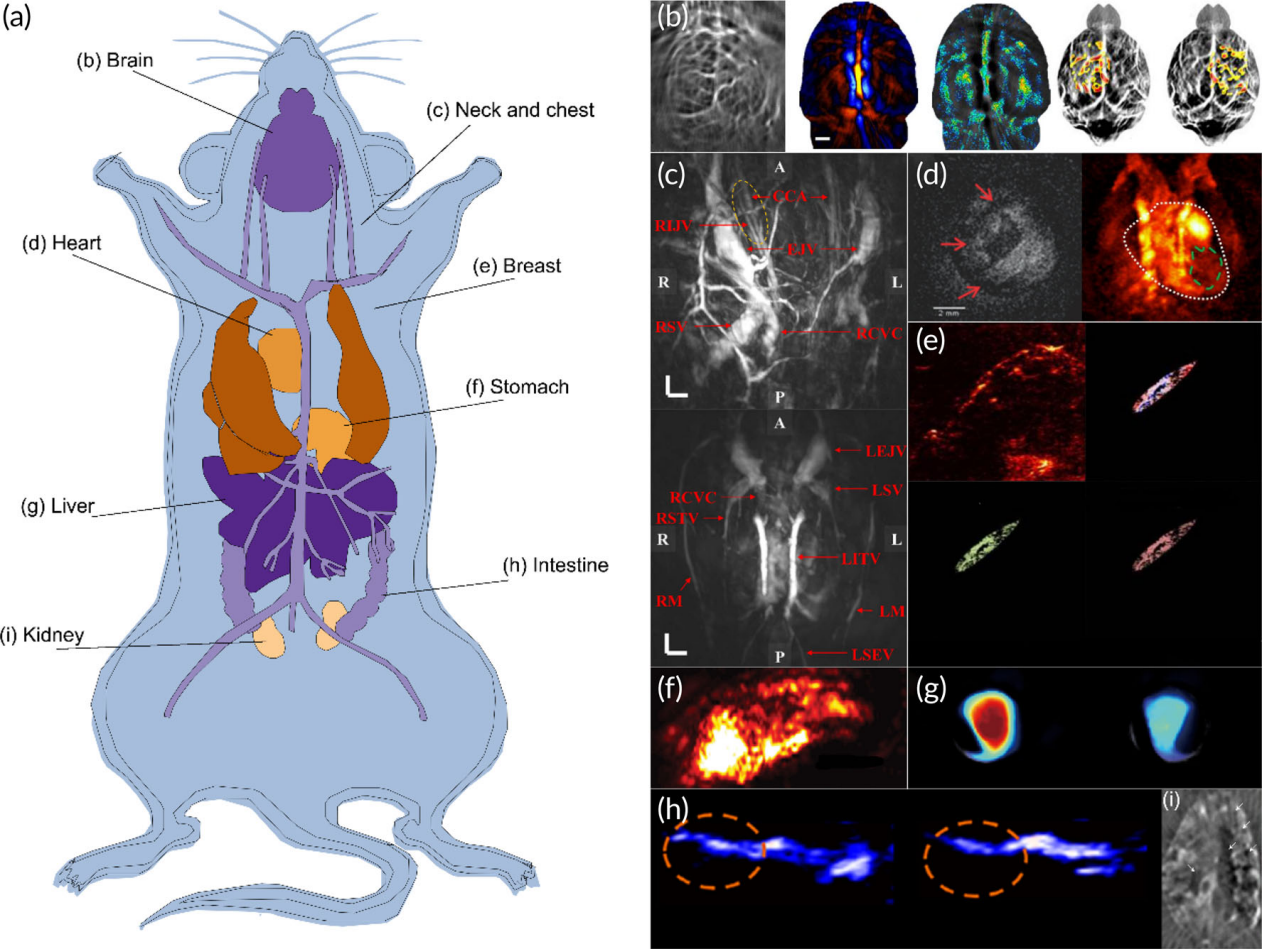
Photoacoustic computed tomography (PACT) images of tissues and organs across the whole body in the rodents. (a) A sketch of the rodent. (b) PACT images of the rodent's brain. Subfigures from the left to the right are a brain vasculature PACT image of a mouse,^92^ a cerebral hemoglobin image of a rat, the corresponding sO_2_ map of the rat,^93^ and PACT images of the cortical responses to forepaw stimulations of a mouse.^10^ (c) PACT images of the rat neck and chest from the top to bottom.^94^ CCA, common carotid artery; EJV, external jugular vein; LEJV, left external jugular vein; LITV, left internal thoracic vein; LM, left mammary; LSV, left subclavian vein; LSEV, left superior epigastric vein; RCVC, right cranial vena cava; RIJV, right internal jugular vein; RM, right mammary; RSV, right subclavian vein; RSTV, right superficial thoracic vein; R, right; L, left; P, posterior; A, anterior. (d) A PACT image of a mouse heart (left),^95^ and a PACT image of a mouse heart with myocardial infarction (right).^96^ (e) PACT images of a mouse mammary gland at multiple wavelengths. Subfigures are raw PACT image, sO_2_ map, lipid map, and hemoglobin map from the upper left to the lower right.^97^ (f) A PACT image of a mouse stomach via nanoprobes.^98^ (g) MSOT images of lipid between healthy (left) and steatotic liver (right) in mice.^20^ (h) PACT images of healthy duodenum (left) and duodenal ulcer (right) in mice via nanoparticles.^97^ (i) A PACT image of the mouse kidney.^99^

With the wide use of model organisms in biomedical and preclinical studies, in vivo tissue imaging and whole‐body imaging are becoming more and more important. PACT has shown great potential in these studies.^4^ To date, PACT imaging has been applied to several different animal models, including mice, rats, pigs, squirrel monkeys, rabbits, dogs, swine, and monkeys.^42^, ^86^, ^87^, ^88^, ^89^, ^90^, ^91^ Taking rodent as an example, PACT can provide whole‐body imaging, as well as in vivo imaging of the brain, chest, breast, heart, liver, stomach, intestine, and kidney of the rodent (as shown in Figure 2).^10^, ^20^, ^92^, ^93^, ^94^, ^95^, ^96^, ^97^, ^98^, ^99^, ^100^, ^101^, ^102^ Using NIR light, PACT can provide whole‐body imaging of animals with acoustically defined spatial resolution.^6^, ^7^, ^12^ Via endogenous hemoglobin contrast, anatomical and vascular structures can be imaged, and by using the wide choice of exogenous optical contrasts, functional, and molecular imaging can be enabled.^45^ Table 2 summarizes the applications of PACT on animal imaging studies. Since a majority of biomedical and preclinical studies have focused on the brain and vascular system, we will provide detailed reviews on PACT imaging of the brain and cardiovascular system in animals. In addition, as there are increasing attentions on adipose tissue, we also review the application of PACT on adipose tissue imaging.

**TABLE 2:** PACT imaging of animals

Animal samples	Results	Significance	References
Brain (mouse, rat, monkey, squirrel monkey)	In 2013, Yao et al. demonstrated that PACT is capable of imaging the metabolic response of a mouse brain to forepaw stimulations using 2‐NBDG as the exogenous contrast and hemoglobin as the endogenous contrast. In 2015, using internal illumination with an optical fiber in the oral cavity, Lin et al. acquired cross‐sectional deep‐tissue images of the rat's brain and the blood vessels in the deep brain in vivo, which was performed on a full‐ring‐array PACT system. In 2016, Nie et al. mapped the cerebral cortex of a monkey brain and quantified the oxygenation saturation in a blood phantom through a monkey skull. In 2019, Zhang et al. imaged the cortical layer of a mouse brain by localizing the centers of single dyed droplets of IR‐780 flowing through blood vessels. They also showed sharper features, resolved finer vascular details, and characterized the droplet flow directions and speeds deep within the brain. In 2021, Chang et al. provided information in 16 mm deep subcortical regions of a squirrel monkey with high temporal resolution via a linear‐array PACT system. In the same year, Lin et al. developed a 3D‐PACT system and visualized whole‐brain vasculatures and hemodynamics in the rat brain.	These studies have proved that PACT can overcome the effect of ultrasound signal attenuation through relatively thick skulls and that animal cerebral cortex imaging is feasible via PACT. By implementing external and internal illuminations simultaneously, PACT can potentially achieve whole‐brain imaging, thereby providing a new way to study the mechanism of cerebral blood supply. Furthermore, the application of PACT as a rapid imaging modality on brain metabolism has been realized.	^10^, ^40^, ^43^, ^86^, ^87^, ^88^, ^92^, ^100^, ^103^
Neck and chest (mouse, rat)	In 2021, Lee et al. obtained PACT images of mouse and rat cervicothoracic vasculature and assessed the vascular branching and structural differences. In the same year, Duan et al. used PACT with a hyperbolic‐array transducer to scan a rat's neck and obtained stereoscopic PA images of the carotid artery noninvasively.	The imaging results of PACT in the neck and chest of animals can be used as a reference for imaging the cervicothoracic vasculature in various preclinical models. PACT imaging of neck and chest can further contribute to noninvasive visualization of the vascular structure and auxiliary diagnosis of vascular diseases in the neck and chest.	^94^, ^102^
Adipose tissue (mouse)	In 2018, Reber et al. employed MSOT to image brown adipose tissue and white adipose tissue in mice via hemoglobin gradients. Brown adipose tissue composition was also analyzed via spectral analysis. In 2021, Chen et al. monitored the reduction of adipose tissues in mice via photoacoustic imaging and protein complexes.	Multispectral PACT technique enables imaging of adipose tissue, analyzing adipose tissue composition and monitoring adipose activations, which provide a new way for studying the physiology of adipose tissue physiology and metabolic diseases.	^104^, ^105^
Heart (mouse, rat)	In 2011, Holotta et al. used PACT to visualize myocardial infarction within ex vivo murine hearts and generated high‐resolution 3D images of the hearts. In 2017, Jing et al. applied PACT imaging of in vivo mouse hearts with myocardial infarction to differentiate lesions from normal areas. Their results suggested that PACT has excellent potential for myocardial infarction diagnosis and lesion staging in cardiology. In 2018, Li et al. used a customized optical fiber‐based PACT to internally illuminate deep targets in phantoms and small animals. They showed that this approach can homogeneously illuminate the surrounding space and substantially enlarge the field of view. They successfully performed noninvasive imaging in rats with myocardial infarction, demonstrating the potential of PACT for the diagnosis and staging of myocardial infarction.	PACT has enabled the visualization of myocardial infarction ex vivo and the generation of high‐resolution 3D images of animal hearts. Due to its improved penetration depth in animal hearts, PACT has great potential for the diagnosis, staging, and monitoring of heart‐related diseases.	^16^, ^95^, ^96^
Breast (mouse)	In 2014, Xi et al. presented a breast imaging technique combining PACT with exogenous nanoparticles as contrast agents, and achieved imaging of breast tumors located as deep as 3.1 cm beneath the normal tissues. In the same year, Wilson et al. achieved detection of concentrations of multiple tissue chromophores including lipid, hemoglobin and sO_2_ in a mouse model of breast cancer. In 2022, Quiros‐Gonzalez et al. applied PACT to quantify hemoglobin concentration and oxygenation in breast tumors, and indicated that PACT can detect response and resistance to bevacizumab.	The results of PACT breast imaging in mice indicated that PACT shows promise in detecting tumors in the breast, and in providing an early indication of the prognosis to antiangiogenic therapy.	^97^, ^106^, ^107^
Muscles (mouse)	In 2016, Lin et al. used PACT to measure the distribution of myoglobin in tissue and the sO_2_ of myoglobin. Then, reported the quantified PA signal of myoglobin sO_2_ based on the dynamics of PACT images at different oxygenation states.	PACT is capable of noninvasive quantification of the sO_2_ of myoglobin changes in the backbone muscle in vivo. Hence, it has great potential for use in sports medicine and for aiding cardiac surgery.	^101^
Stomach and intestine (mouse)	Li et al. and Huang et al. separately achieved functional gastrointestinal imaging combined PACT with pH‐responsive nanoparticles.	PACT with pH‐responsive agents show promise in quantitative diagnosis of gastrointestinal diseases.	^64^, ^98^, ^108^
Liver (mouse)	MOST has played a major role in liver imaging. In 2014, Taruttis et al. used MSOT to image the uptake of indocyanine green in the liver. In 2018, Wu et al. combined MSOT with two exogenous optoacoustic probes, and localized drug‐induced liver injury and metastatic tumors in mice. In 2021, Huang et al. employed MSOT to detect lipids and monitor the kinetics of indocyanine green in mouse livers, and to stratify hepatic steatosis.	The results indicated that MSOT was an efficient tool for liver imaging in preclinical studies. Multispectral PACT technique may potentially be applied for the diagnosis and assessment of liver diseases.	^20^
Kidney (mouse, piglet)	In 2012, Muhammad et al. used RC‐PACT to determine the anatomy and glucose uptake of mouse kidney tumors with a single modality. In 2016, Bo et al. confirmed the sensitivity of PACT in the detection of cysts by in vivo and ex vivo imaging of mice with kidney disease and healthy kidneys. In 2020, Li et al. completed tissue model imaging and in vivo piglet kidney vascular imaging at a depth of 10 cm using PACT with optimized fiber diffusers.	Successful visualization of kidney cysts in mice via PACT facilitates renal volume measurement, nephrotic tissue classification, and cyst extraction. PACT can also realize real‐time label‐free imaging of renal vasculature, which can be used to monitor renal vascular injury and reduce the damage of large hematoma to relevant tissues.	^9^, ^42^, ^99^
Whole body (mouse)	In 2012, Xia et al. used RC‐PACT to obtain a series of cross‐sectional images of multiple organs across the entire mouse with high imaging speed, and they used endogenous hemoglobin contrast to show organ vessels clearly. Two years later, they succeeded in reconstructing clearer vascular and anatomical images by capturing animal respiratory waveforms during acquisition and classifying the PACT images according to different respiratory stages. In 2017, Li et al. developed SIP‐PACT and demonstrated its ability to provide noninvasive imaging of mouse anatomy in real time, clearly showing suborgan vasculature and structures. In addition, they mapped the whole‐body arterial network.	Whole‐body PACT can obtain anatomical structures and detailed images of the vascular structures within organs. Moreover, whole‐body PACT images with improved image quality after respiratory gating can be used to monitor the pathological changes of internal organs, thus providing a promising approach to whole‐body functional and metabolic imaging.	^6^, ^7^, ^12^

Abbreviations: PACT, photoacoustic computed tomography; RC, ring‐shaped confocal; SIP, single‐impulse panoramic.

### 3.1 PACT imaging of animal brain

The main application of PACT in model animals is brain imaging. Since Yang et al. imaged a monkey head sample model through a 2 mm thick skull and successfully reconstructed the major vasculature of the cerebral cortex in 2008, the application of brain PACT imaging of small‐ and medium‐sized animals has been continuously improved and updated.^86^ However, due to the limited light penetration depth, most studies have focused on imaging the cortex. It was only in 2015 that Lin et al. improved the imaging method to achieve the first deep‐brain structural imaging of rats in vivo.^43^ Afterward, Lu et al. and Zhang et al. separately surpassed the acoustic diffraction limit by using exogenous nanoparticles and dyed droplets, as displayed in the left subfigure of Figure 2b.^92^, ^103^ Compared to imaging with hemoglobin as an endogenous optical contrast chromophore, exogenous agents can construct super‐high‐resolution images to show capillaries in deep tissues, which is more beneficial for hemodynamic studies in the mouse brain cortex. Except for blood flow speed, PACT can measure other parameters required for quantifying cerebral metabolic rate of oxygen, namely vessel cross section, sO_2_, total concentration of hemoglobin, and tissue volume.^10^

In addition to its rich applications in displaying brain structure, vascular networks, and hemodynamics, PACT also excels in functional and metabolic brain imaging (the middle two subfigures of Figure 2b).^93^, ^103^ Back in 2013, using 2‐NBDG as the exogenous contrast and hemoglobin as the endogenous contrast, Yao et al. demonstrated that PACT is capable of imaging the metabolic response of a mouse brain to forepaw stimulations, as shown in the right subfigures of Figure 2b.^9^ Tang et al. also demonstrated that PACT could be used for monitoring the cerebral responses in behaving rats to sensory stimulus.^93^ Later, researchers confirmed noninvasive, unlabeled, and functional PACT in rat brain for accurately mapping brain injury and cerebral hemodynamics.^13^, ^112^, ^113^ Li et al. and Nasiriavanaki et al. independently achieved functional connectivity calculation from the rodents' brain using the oxygenation dynamics recorded from the PACT, which could only be achieved by blood oxygenation level dependent (BOLD) functional magnetic resonance imaging (fMRI) previously.^6^, ^114^ Recently, based on tau‐targeted exogenous contrast agent, tau protein imaging in the whole mice brain was achieved via MSOT, which had potential use in detecting tau spreading and clearance in tauopathy‐related neurodegenerative diseases.^21^

**FIGURE 3:**
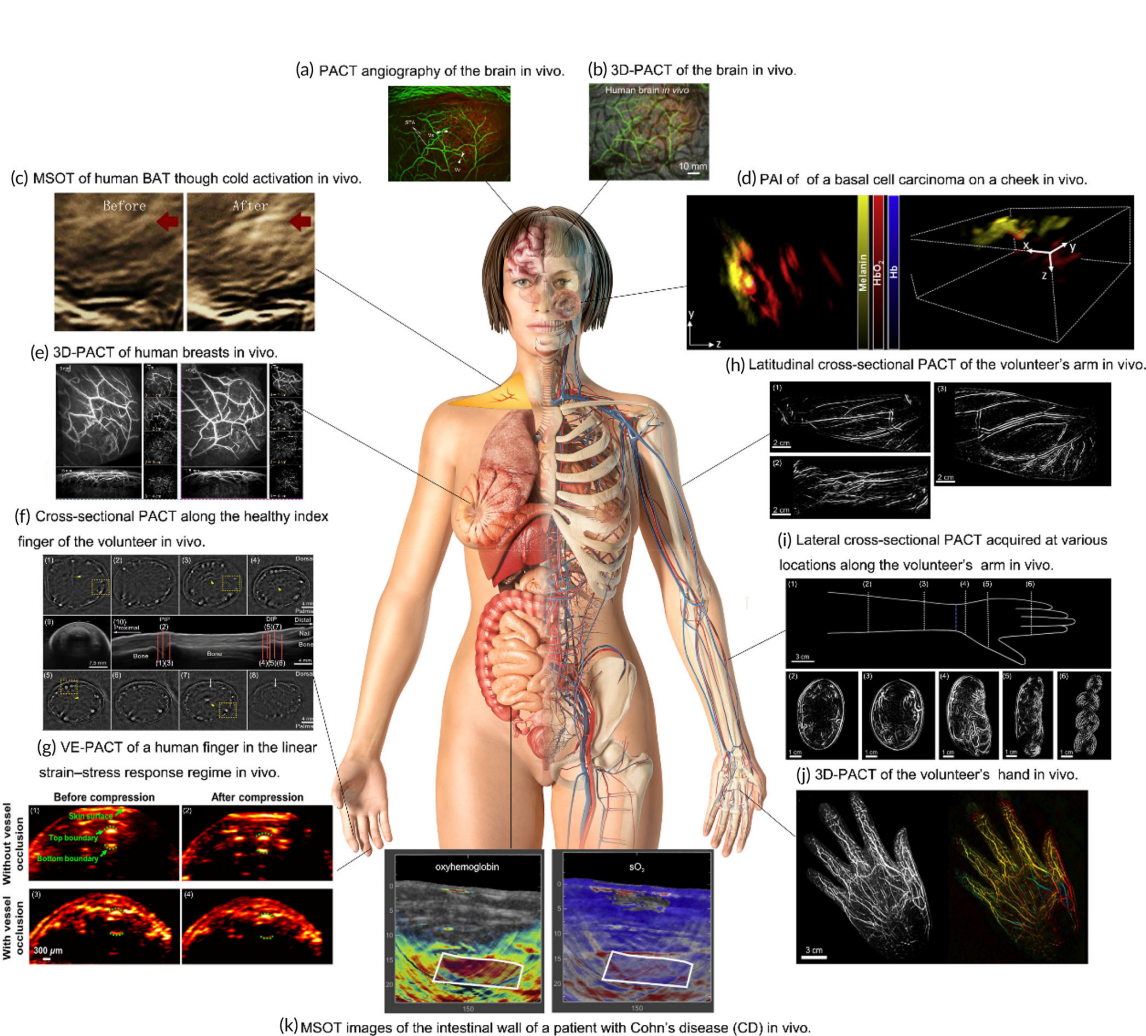
Photoacoustic computed tomography (PACT) imaging of various organs and parts of the human body. (a) PACT angiography of the brain in vivo.^34^ Images are segmented into scalp (green) and cortical (red) regions. (b) 3D‐PACT image of the brain in vivo.^109^ (c) Multispectral optoacoustic tomography (MSOT) images of human brown adipose tissue (BAT) before and after cold exposure.^104^ (d) PACT image of a basal cell carcinoma on the cheek skin with cross‐sectional view on the left and 3D rendering of the lesion on the right.^120^ (e) 3D‐PACT images of human breasts in vivo.^100^ The upper left figure is a fluoroscopic angiogram of the right breast in a healthy person, the lower left figure is a maximum amplitude projection (MAP) image of the right breast in lateral view, and the four smaller images on the left are cross‐sectional images of the right breast from the nipple to the chest wall in different coronal planes. Each cross‐sectional image is a 1 cm thick MAP of the breast section. The right figures are the left breast of the same person. (f) Cross‐sectional PACT images along the healthy index finger of the volunteer in vivo, which show the blood vessels along the length of the finger as the intense white areas.^25^ Subfigures (1)–(8) show eight cross sections, containing images across the proximal interphalangeal point joint, distal interphalangeal point joint, and the nail wall, in approximately the same locations as those marked in the ultrasound images. (g) Vascular elastic PACT images of a human finger in linear strain–stress response regime in vivo.^110^ Subfigures (1) and (2) show comparative cross‐sectional PACT images of a human finger without vascular occlusion before and after compression, and subfigures (3) and (4) show corresponding PACT images with vascular occlusion before and after compression. (h) Latitudinal cross‐sectional PACT of a volunteer's arm in vivo.^29^ The three images are longitudinal cross sections of the upper right forearm, the lower right forearm (ending at the wrist), and the right biceps muscle of the volunteer. (i) Lateral cross‐sectional PACT images acquired at various locations along a volunteer's arm in vivo.^29^ (1) A sketch image of the arm. The white dotted line details the location of each cross‐sectional image in images (2)–(6). The blue dotted line marks the wrist, where the carpal bone meets the radius and ulna. (2) An image of the upper forearm, 10 cm above the wrist. (3) An image of the lower forearm, 3 cm above the wrist. (4) An image of the palm, 1 cm below the wrist. (5) An image of the middle of the palm, 3 cm below the wrist. (6) An image of the finger (excluding the thumb), approximately midway between the proximal phalanges. (j) 3D‐PACT images of the volunteer's hand in vivo.^29^ The MAP of the PA signal along the Z‐axis is presented on the left, and a color‐coded depth image of the hand is presented on the right. (k) MSOT images with increased signals for oxyhemoglobin and sO_2_ in the intestinal wall of a patient with Cohn's disease.^111^

### 3.2 PACT imaging of animal cardiovascular system

Cardiovascular imaging is another major application of PACT. Compared with the low tissue contrast of ultrasound imaging, poor spatial resolution and radiation exposure of single‐photon emission computerized tomography, and strict requirements for MRI, PACT shows unique advantages in cardiovascular imaging with excellent light absorption contrast, super‐high resolution, and deep penetration.^96^ Researchers achieved PACT imaging of vasculature in the chest (Figure 2c), demonstrating the potential of PACT in the assessment and diagnosis of cardiovascular disease.^94^, ^102^ In addition of vascular imaging, heart imaging has been realized. In 2011, Holotta et al. performed PACT imaging on nine ex vivo cardiac models of mice with different infarct survival periods (the left subfigure of Figure 2d). Reconstructed images showed high agreement with MRI and histology, which demonstrated the ability of PACT to visualize myocardial infarction in vitro.^95^ A few years later, Jing et al. developed a PACT platform to monitor deep tissues in mouse heart in vivo and noninvasively imaged rodent hearts with myocardial infarction (the right subfigure of Figure 2d), which suggested that PACT has excellent potential for myocardial infarction diagnosis and lesion staging in cardiology.^96^ Later, Li et al. optimized the PACT hardware system by combining internal light illumination with external ultrasound detection to improve the penetration depth of PACT in imaging deep organs of large animals and humans. They demonstrated that the improved system can image the beating hearts of mice under multiple overlays and makes it possible to image internal organs that are deeply seated in the human body and relatively close to body cavities.^16^

### 3.3 PACT imaging of animal adipose tissue

Recently, the study of adipose tissue has attracted increasing attention, due to the association between adipose tissue with a variety of diseases, including obesity, diabetes, and cachexia.^20^ PACT, as a hybrid imaging modality with strong optical contrast and high spatial resolution, has been used in adipose tissue imaging. In an ex vivo study, Reber et al. demonstrated that PA signal of brown adipose tissue (BAT) was much higher than that of white adipose tissue in the NIR range using the MSOT.^104^, ^105^ An in vivo research of mouse adipose tissue showed that MSOT can image BAT via MSTO and hemoglobin gradients. The research further imaged BAT in the resting state and in activation via norepinephrine. Moreover, the composition of adipose was also revealed via hemoglobin, water, and lipid signal from MSOT.^104^ Recently, Cheng et al. detected the complex changes of adipose tissue induced by treatment, including activity, lipid catabolism, and angiogenesis via photoacoustic imaging and a protein complex.^105^

## 4 THE APPLICATION OF PACT IN PRECLINICAL AND CLINICAL HUMAN BODY IMAGING

### 4.1 PACT imaging of human body

Various preclinical experiments have shown that PACT can achieve high‐resolution optical contrast imaging in a variety of biological tissues with penetration depth up to several centimeters, making PACT a promising technique for preclinical and clinical imaging applications in various human organs. PACT imaging of human body mainly uses endogenous substances such as hemoglobin, melanin, lipids, and proteins as optical absorption substances.^115^ Some exogenous contrast agents such as indocyanine green and methylene blue can be used to enhance the performance of PACT imaging of human body.^116^, ^117^, ^118^ With noninvasive, label‐free, and radiation‐free features, PACT is friendly to patients, medical staff, and clinical application. Recent trending and relatively well‐established applications include breast imaging, dermatological imaging, vascular imaging of the extremities, carotid angiography, gastrointestinal imaging, and adipose tissue imaging.^115^

PACT imaging of various organs and parts of the human body has been demonstrated in numerous studies (as shown in Figure 3). Previous studies have documented the application of PACT in animal brain structural and functional imaging.^10^, ^43^, ^86^, ^87^, ^88^, ^92^, ^93^, ^100^, ^103^ However, in human brain, the acoustic aberration resulted from the skull has hindered PACT imaging of human brain.^109^ Until recently, Na et al. performed cerebral vascular imaging of volunteers using baseline PACT, as shown in Figure 3a.^34^ Later, Na et al. developed high‐speed 3D‐PACT and used it to perform functional imaging of the human brain, as shown in Figure 3b. They used massively parallel ultrasound sensors arranged around the human head to obtain tomographic images of the brain. From these images, they successfully measured deoxyhemoglobin, oxygen, and hemoglobin concentrations and quantified saturation and blood volume. They proved that high‐speed 3D PACT can detect functional activation faster than BOLD‐fMRI, so it is feasible for functional PACT of the brain.^109^

In terms of adipose tissue imaging, PACT has demonstrated its feasibility for imaging BAT morphology, composition and longitudinal activity in mice.^104^, ^105^ For human body, Buehler et al. imaged the lipomas at different areas of six subjects used a clinical MSOT system.^119^ Clinical MSOT can also image BAT based on hemoglobin gradients, and can detect BAT activation after cold exposure by the increase of PA signal, as shown in Figure 3c.^104^ Owing to the easy accessibility of human skin, PACT imaging of skin‐related diseases, especially skin cancer has gained intense attention. As displayed in Figure 3d, Chuah et al. acquired 3D volumetric images of basal cell carcinoma via clinical MSOT.^120^ Breast PACT imaging has attracted huge research interest, due to the fact that breast is easy to cover by PACT. Lin et al. reconstructed a fluoroscopic angiogram of a healthy human breast, as shown in Figure 3e.^100^ They achieved in vivo imaging with a penetration depth of 4 cm and an almost isotropic spatial resolution of 0.37–0.39 mm by scanning the breast in a SBH of 10 s.

As a majority of preclinical and clinical PACT imaging studies have focused on the extremities, skin, and breast, we will provide detailed reviews on PACT imaging of relevant regions in the following sections. In addition, we will also review the application of PACT imaging of the digestive tract.

#### 4.1.1 PACT imaging of extremities

The flexibility and small size of the human extremities, the presence of capillaries, and the low penetration depth required make the extremities, especially the hands convenient targets for preclinical PACT imaging. In 2010, Hu et al. used Fabry–Pérot interferometer‐based PACT to image microvessels located 4 mm below the skin on the palm.^121^ In 2014, Es et al. developed a PACT device to obtain cross‐sectional images of the finger and used it to study the fingers of healthy subjects. The device focused on visualizing the digital vasculature and observing the vascularity of the two interphalangeal joints and showed the first PA images of vascularized rich human fingers. Figure 3f shows a series of photoacoustic cross‐sectional images obtained by Es et al. along the index finger of a volunteer during a real‐time scan.^25^ In 2015, Biswas et al. reconstructed the PA signal from the epidermis of the hand to reduce the bad effects on the identified bone surface. This technique identified not only artifacts but also the interphalangeal joint space, helping to find inflamed synovial membranes and locate areas of interest.^26^ At the end of the same year, Hai et al. applied PACT to quantitatively assess the vascular elastic (VE) properties in human hands in vivo. In addition to the information that PACT imaging can provide (structure, flow rate, molecular concentration, and sO_2_ level of hemoglobin), VE‐PACT adds important elasticity information, confirming the fact that the vascular compliance of human fingers is reduced due to vascular occlusion. Figure 3g shows VE‐PACT images of a human finger during a linear strain–stress response system implemented by Hai et al.^110^ In 2019, Wray et al. used PACT to image the vascular network across a volunteer's arm in vivo, as shown in Figure 3h.^29^ They also acquired lateral cross‐sectional images at different locations on the right arm of the volunteer, as shown in Figure 3i, and reconstructed a 3D PACT image of the volunteer's right hand, as displayed in Figure 3j.^29^

#### 4.1.2 PACT imaging of human skin

Since the human skin is easily accessible, PACT has been widely used in many preclinical and clinical dermatologic scans. Due to the unprecedented sensitivity of PA signals to hemoglobin and melanin, PACT has been commonly used for imaging the subcutaneous vascular system and melanoma.^47^ PACT can provide high‐resolution sO_2_ measurements using hemoglobin as an endogenous contrast agent, which is significant because sO_2_ is an important biomarker for skin cancer healing, treatment monitoring, and wound healing monitoring.^48^ Back in 2010, Favazza et al. imaged sO_2_ during arterial occlusions created by an inflated arm cuff.^48^ In 2016, Hsu et al. even resolved sO_2_ to the single cell level in the human stratum corneum.^49^

Other skin conditions such as psoriasis, dermatitis, and port wine stains (PWSs) can also be quantitatively detected and assessed by PACT.^47^ PWS is a reddish birthmark that can appear anywhere on the body surface, and because it is associated with veins concentrated in the superficial dermis of the affected area, PACT imaging is useful in monitoring PWS.^122^ Viator et al. developed a PACT imaging system to study excess vascular and capillary malformations in PWS and to quantify PWS thickness for laser treatment planning. The study further found that with the help of PACT, laser treatment could be appropriately adapted to remove as much PWS as possible while still preserving the epidermis.^123^ In 2018, Ossadnik et al. found that capillary rings, which curl around papillary vessels and are tightly wrapped by the epidermis, could be served as powerful indicators of early psoriasis angiogenesis and could be accurately identified by highly sensitive PACT, revealing the great potential of PACT in early psoriasis diagnosis.^124^ In 2020, Hindelang et al. began exploring PACT imaging in dermatitis and developed a PACT‐based patch.^125^

Assessing burns and monitoring the healing process are other aspects for PACT dermatologic imaging.^126^ In 2012, Tsunoi et al. used PACT to map the distribution of photosensitizers in the skin to ensure adequate diffusion throughout the infected area.^127^ For acute burns, Ida et al. used PACT to clearly delineate the boundary between edematous coagulated burn tissue and healthy perfused tissue.^128^ In addition to providing diagnostic assistance, Später et al. used PACT to monitor the healing process of burns or other skin wounds by studying the density of lipid microvascular fragments required to reconstruct host blood vessels.^126^ Although skin biopsy is currently the gold standard for the diagnosis of dermatological diseases, due to its limitations, such as its inability to assess vascular function and high sensitivity to biopsy time and age of lesions, noninvasive imaging techniques with sufficient penetration to scan skin tissue, such as PACT, will be the first choice for the diagnosis of dermatological diseases in the future.^47^

#### 4.1.3 PACT image of human digestive tract

Furthermore, PACT has been applied to image the digestive tract. In 2016, via MSOT imaging of hemoglobin and sO_2_, Waldner et al. assessed functions of the colon in patients with Cohn's disease, and found increased hemoglobin and sO_2_ values in the intestinal wall in patients with Cohn's disease (Figure 3k).^111^ In 2018, Wang et al. completed human swallowing imaging using baked barley as a contrast agent.^15^ In late 2019, Yang et al. achieved the first application of ultrasonic co‐orbital PACT in the human distal digestive tract. The spectral features, image features, and standard deviations of the mean radon transform provided by PACT can assist existing radiological techniques in the diagnosis, management, and monitoring of colorectal cancer.^115^

### 4.2 Tumor imaging

PACT can provide morphological and functional information on early stage cancer using endogenous or exogenous contrast agents.^129^ Angiogenesis plays an essential role in the development, invasion, and metastasis of cancer, which results in a locally abnormal vascular morphology and increased density. Structural and/or functional vascular changes and associated deficits in oxygenation are crucial indicators of early stage cancer and potential targets of relevant therapy.^130^ PACT, as a powerful tool for label‐free angiography, can achieve quantitative analysis of the spatial distribution, morphologic changes, and density of vessels, making it an effective approach for tumor imaging.^130^ In addition, functional features such as blood flow velocity and blood sO_2_ can also be obtained with PACT.^129^

To date, in terms of animal models, deep tumor imaging, monitoring the effect of certain therapies have been realized,^65^, ^131^ and measurements of tumor microenvironments, including pH change, enzyme activity, and reactive oxygen species levels have been achieved.^131^ As for tumors in human body, there have been several PACT‐based tumor imaging studies involving breast, melanoma, prostate, thyroid, ovarian, and cervical cancers, and the studies are summarized in Table 3. Since PACT imaging of skin cancer and breast cancer received much research attention, we will give a comprehensive review of these aspects in the following sections.

**TABLE 3:** Summary of tumor imaging studies based on PACT

Regions	Research details
Breast cancer	Increases in vascular density and changes in blood sO_2_ are key markers for breast malignancies.^129^ Enabled by rapidly toggling laser wavelengths in the NIR spectral range, PACT is capable of whole breast functional imaging, yielding clear images of breast vessels and tumors simultaneously.^132^ In addition to vascular density imaging, detecting relative changes of tissue components caused by tumors is another advantage of PACT for breast cancer screening.^129^ By analyzing changes in the ratios of different forms of hemoglobin and the relative blood oxygenation, the spatial heterogeneity of a breast tumor can be quantified.^133^ A recent study achieved volumetric imaging of human breast cancer in 15 s using the SBH‐PACT system, with the real‐time acquisition of 2D slices of breast anatomy, and demonstrated PA angiography with no breathing‐induced motion artifacts.^22^ PACT is able to provide depth‐encoded PA vessel images of cancerous breast tissue showing the detailed vascular structure, and it is also able to present images of quantitative hemoglobin concentration and sO_2_ in healthy and afflicted breasts to accurately visualize tumor regions.^22^, ^134^
Skin cancer	Due to its inherent advantages, PACT holds great promise for skin cancer detection and quantification.^135^ PACT uses endogenous tissue chromophores (e.g., melanin or hemoglobin) to provide a unique combination of optical absorption contrast and ultrasonic spatial resolution for attaining high‐sensitivity images at centimeter‐level depths. Therefore, in vivo images of deeper structures can be provided, enabling determination of tumor thickness and visualization of blood flow in skin vasculature.^136^ In addition to being used for deep‐tissue imaging and determining the thickness and structure of melanoma, PACT can be used to visualize lymph node metastases and detect circulating melanoma cells, allowing for the clinical evaluation of primary and metastatic malignant melanoma, noninvasive visualization of tumor boundaries, and assistance with the assessment of metastatic status, which can facilitate more effective treatment and better clearance of tumor cells and reduce the need for additional biopsies.^50^
Prostate cancer	With structural, functional, and molecular imaging characteristics, PACT is expected to be an effective imaging modality for the early diagnosis and treatment evaluation of prostate cancer.^72^ In an ex vivo study, PACT was verified to differentiate malignant prostate tissue, benign prostatic hyperplasia, and normal human prostate tissue from prostate tissue sections by indicating the presence of deoxyhemoglobin, oxyhemoglobin, lipids, and water.^137^ In vivo studies have demonstrated the feasibility of PACT for recognizing the angiogenesis of prostate cancer based on how the intensity of PA signals corresponds to vascular parameters (e.g., vessel length and vascular density). Therefore, PACT can be potentially applied for imaging prostate cancer angiogenesis by comparing the microvascularity between normal prostate tissue and cancerous prostate tissue.^138^, ^139^
Thyroid cancer	Multispectral PACT has been performed on surgically excised thyroid tissue, and chromophore images representing the optical absorption of deoxyhemoglobin, oxyhemoglobin, lipids, and water have been reconstructed. Chromophore analysis on PA images can aid in differentiation of malignant from nonmalignant thyroid tissue with a sensitivity of up to 70%.^140^, ^141^, ^142^
Ovarian cancer	Oxyhemoglobin saturation, an indicator of tumor metabolism and therapeutic response, is also an important diagnostic measure of PACT. In cystic ovarian tissue, the PACT penetration depth may reach 6–7 cm, which enables imaging of more than 95% of the ovaries.^143^ PACT can show the distribution of deoxyhemoglobin and oxyhemoglobin, contributing to the identification of both high‐grade epithelial ovarian cancer and low‐grade ovarian tumor.^144^
Cervical cancer	PACT is capable of intact scan both around the external orifice and in the cervical canal. In one previous study, depth MAP images obtained through in vitro experiments were analyzed to evaluate the extent of angiogenesis for different clinical stages of cervical cancer. The results revealed that the mean optical absorption of the cervical lesions was closely related to the severity of cervical cancer.^145^ With the ability to distinguish cervical lesions at the depth of 5 mm, PACT may have great utility in the clinical diagnosis of cervical cancer.^145^
Colorectal cancer	PACT can be used to differentiate normal from tumor tissues in the colorectum through quantitative measurement of hemoglobin concentration and the spectral features. A pilot study reported PACT imaging of colorectal cancer samples ex vivo, and achieved area under the curve values of more than 0.95 in distinguishing normal from tumor tissues.^30^

Abbreviations: NIR, near‐infrared; PACT, photoacoustic computed tomography; SBH, single breath‐hold.

#### 4.2.1 PACT imaging of skin cancer

Skin and subcutaneous tissue are easy to assess for PACT. PACT imaging of skin and subcutaneous tissues reveals vascular structure, oxygenation, and flow. These parameters can provide information about the tumor microenvironment, such as angiogenesis and hypoxia, which are of clinical importance to the detection and assessment of skin cancer.^129^ Among various skin cancers, melanoma is the most aggressive skin cancer.^91^, ^129^ Melanoma initially grows within the epidermis, which is followed by a vertical growth phase with deeper extension.^146^ Therefore, noninvasive and quantitative depth assessment for melanoma is of clinical importance.^129^ In addition, early detection of malignant melanoma and subsequent precise surgical extirpation can reduce the mortality of this fatal cancer.

**FIGURE 4:**
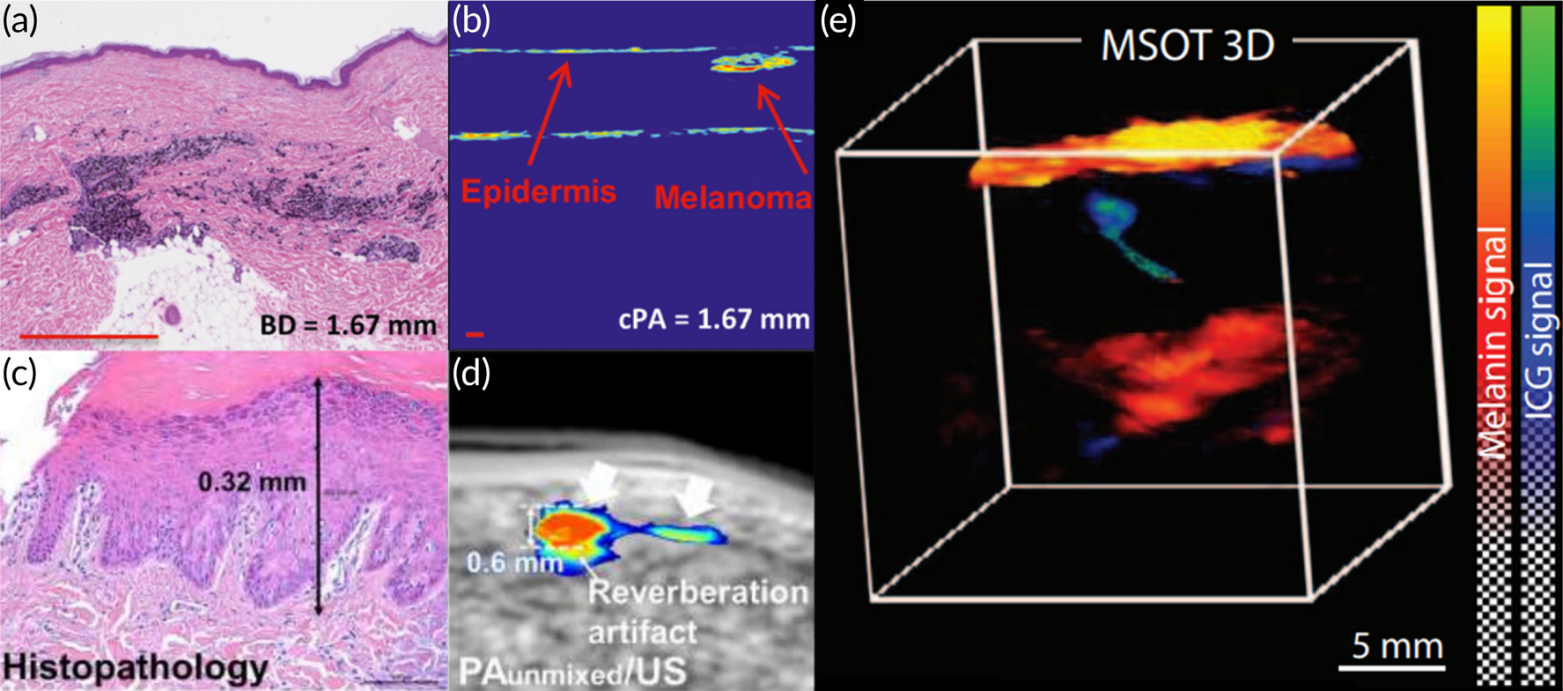
Photoacoustic computed tomography (PACT) imaging of melanoma. (a,b) Histological and corresponding PACT images of the melanoma imaged with the linear array‐based handheld photoacoustic probe.^146^ (c,d) Histological and corresponding 3D multispectral optoacoustic tomography (MSOT) images of the melanoma. Specifically, (d) is the unmixed spectral PA image.^147^ (e) A rendering map of sentinel lymph node with melanin and indocyanine green signal, demonstrating the metastatic melanoma in the sentinel lymph node.^148^

PACT has excellent capabilities for melanoma detection and depth quantification. Kim et al. reported the results of a cutaneous melanoma lesion excised from the heel of a patient with a size of approximately 1.5^2^ in. Three‐dimensional reconstructed photoacoustic images of the lesion matched well with pathologic examination.^135^ In addition, both linear array‐based handheld photoacoustic probe and 3D MSOT system were used to image melanoma (Figure 4a–d), and the findings demonstrated that the PACT‐based skin depth was more accurate than the temporary incisional biopsy depth based on histological findings.^91^, ^146^, ^147^ Another promising application in human melanoma imaging via PACT is the detection of metastatic status of sentinel lymph nodes. In a relevant study, MSOT was used to image sentinel lymph nodes in melanoma patients ex vivo. The results revealed a significant improvement in the detection of metastases compared to lymph node excision protocols. The detection depth of the sentinel lymph nodes was further improved to 5 cm using indocyanine green as the contrast agent (Figure 4e).^148^

In addition to melanoma, there were studies on nonmelanoma skin cancer via PACT. For instance, as displayed in Figure 3d, Chuah et al. quantified tumor dimensions of basal cell carcinoma via the MSOT, and reported high correlations between tumor dimensions detected by MSOT and histopathological examination.^120^ These pilot studies support the development of PACT as a preoperative screening tool to guide skin cancer resection. Although there are still technical limitations and a lack of standardization of clinical equipment, the integration of a portable, tunable, multiwavelength, fast‐scanning PACT system could be expanded to a variety of preclinical and clinical skin cancer applications.^129^

#### 4.2.2 PACT imaging of breast cancer

Breast cancer is one of the most commonly diagnosed cancer in women.^127^ Compared with other body parts, the breast has lower vascular density. The dense breast tissue has little effect on PA signals.^134^ In addition, angiogenesis and changes in blood sO_2_ and hemoglobin concentration are hallmarks of breast malignancy.^130^, ^134^ PACT can visualize blood vessels, quantitatively assessing angiogenesis, blood sO_2_, and total hemoglobin concentration, making it an ideal method for breast cancer detection.^129^, ^134^ To date, PACT‐based breast cancer imaging has gradually moved from research to clinical practices, with several clinical PACT breast imaging systems.^134^

**FIGURE 5:**
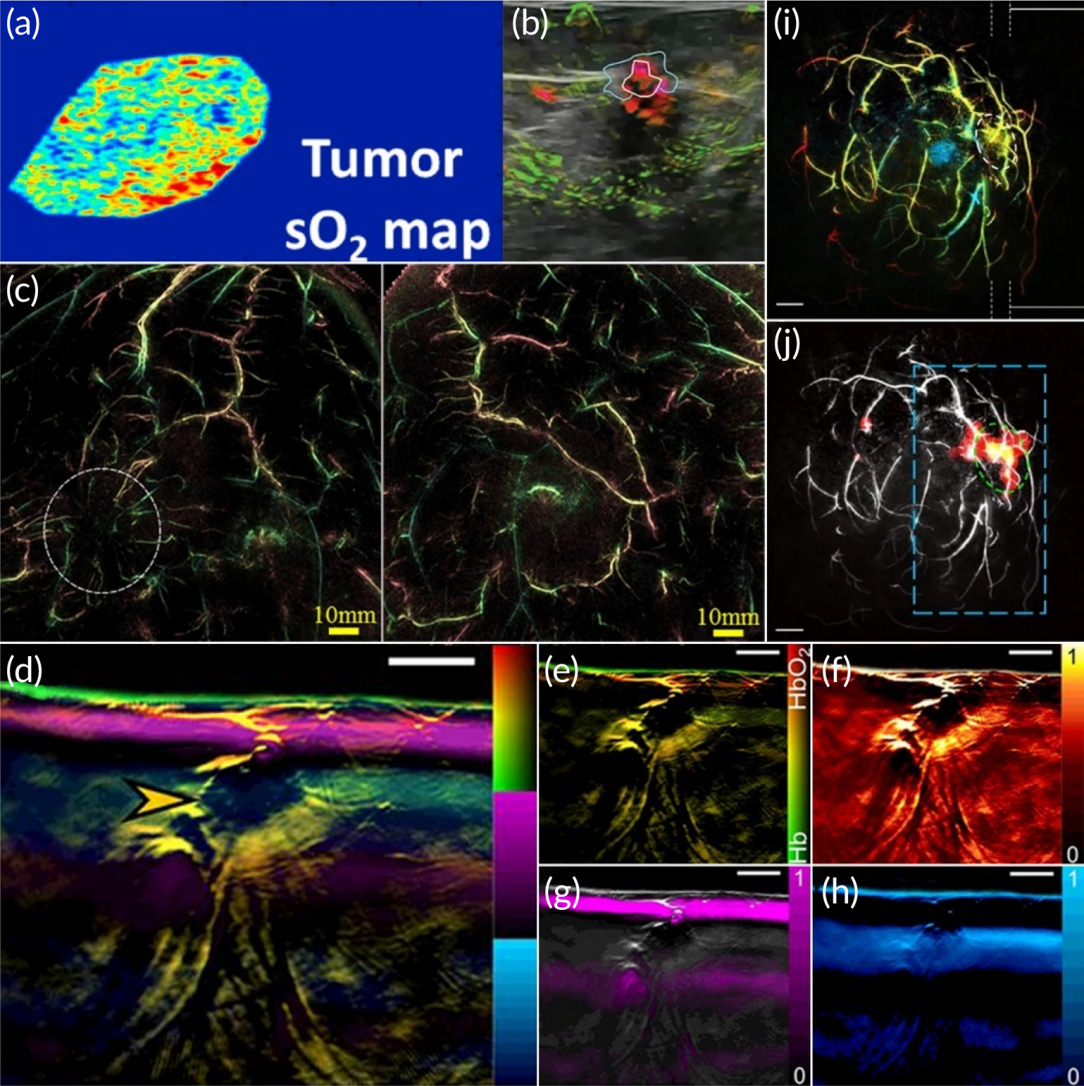
Photoacoustic computed tomography (PACT) imaging of breast cancer. (a) Calculated sO_2_ map of the breast tumor via PACT.^149^ (b) Ultrasound and PACT combined images of invasive mixed ductal and lobular carcinoma in the breast, with oxygenated blood as green and relatively deoxygenated blood as red.^150^ (c) PACT images (maximum intensity projection) of the breast tumor (left) and the contralateral side (right). The location of the tumor was indicated by the dashed circle. Specific structure of blood vessels could be seen around the tumor, whereas not in the contralateral side.^151^ (d) Unmixing multispectral optoacoustic tomography (MSOT) image of the breast tumor showing the four key absorbers.^133^ (e) MSOT image of hemoglobin and oxyhemoglobin. (f) MSOT image of total blood volume. (g) MSOT image of lipid. (h) MSOT image of water content. (i) Single breath‐hold (SBH)‐PACT angiograms of invasive ductal carcinoma in the breast.^22^ (j) Automatic tumor detection on vessel density maps by SBH‐PACT.^22^

In 1994, the idea of using photoacoustic for breast imaging was proposed, and the first experiment was implemented in 2001.^134^ Since then, many PACT configurations have been reported.^134^ In summary, most of the existing PACT configurations used NIR‐I or NIR‐II lasers to identify features including sO_2_ (Figure 5a), hemoglobin concentration (Figure 5b),^149^, ^150^ and to visualize vessel density, vasculature in the breast for tumor detection (Figure 5c).^132^, ^151^, ^152^ In addition to morphological imaging, detection of relative changes in tissue composition due to tumor is another advantage of PACT in breast cancer screening.^129^ Via the MSOT configuration, Diot et al. performed a pilot study in which four different tissue components of breast tissue were precisely separated, and by analyzing the ratio of different forms of hemoglobin and relative blood oxygen content and other changes, the spatial heterogeneity of breast tumors can be further quantified with significantly higher estimates of hemoglobin in both the tumor center and tumor margins (Figure 5d–h).^133^ The recently developed SBH‐PACT enables volumetric imaging of human breast cancer in less than 15 s, presents two‐dimensional cross‐sectional section images in real time, allows PA angiography without motion artifacts caused by respiration.^22^ The SBH‐PACT provides depth‐encoded photoacoustic angiograms of the cancerous breast with detailed vascular structures, and quantitative images of hemoglobin concentration and sO_2_, whereby tumor areas are visualized (Figure 5i,j).^22^ In addition, the system can monitor treatment response to neoadjuvant chemotherapy by acquiring information similar to contrast‐enhanced MRI, but with higher spatial resolution and faster imaging speed, opening up new capabilities for real‐time imaging and clinical practice.^22^, ^134^

The application of PACT for tumor detection is not limited to the abovementioned scenarios, and it is moving toward clinical translation. Accurate localization and characterization of tumors are crucial to provide timely intervention and effective treatment. PACT is suitable for multidimensional imaging of anatomy, molecules, metabolism, and genetics in biologic specimens, enabling tumor diagnosis from macroscopic to microscopic scales. It promises to improve early detection of cancers with resultant improvements in survival rates.^129^

## 5 CONCLUSION AND OUTLOOK

As a noninvasive hybrid imaging modality, PACT has a wide range of applications in biomedical research. As tissues vary greatly in their sensitivity to light of different wavelengths, PACT uses this endogenous tissue contrast to highlight imaging targets.^115^ For instance, the hemoglobin in erythrocytes is sensitive to visible light with wavelengths ranging from 500 to 650 nm, so hemoglobin is used as the optical absorber for vascular imaging. Melanin is sensitive to visible and NIR light in the band around 750 nm, so it is used as an optical contrast agent for melanoma.^4^, ^22^, ^23^ In addition, it has been revealed that DNA and RNA in the nucleus have strong optical absorption at 200–300 nm ultraviolet light, whereas water and lipids have characteristic absorption peaks at 900–1200 nm in the far‐ and mid‐infrared regions.^129^ These materials have the potential to be used as endogenous contrast agents in biomedical PACT imaging. Ever since the idea of using photoacoustic technique to image biological tissues, many PACT configurations have been developed, such as MSOT, SBH‐PACT, and so forth.^22^, ^28^ Based on the review of existing research studies, we think that there are two major development directions for PACT in the future, one is the use of exogenous agent, the other is the development of imaging technique.

Endogenous contrast agents are mainly used for structural imaging of tissues, particularly anatomical and vascular imaging of tissues.^7^, ^12^ Advanced functional imaging and molecular imaging require the use of exogenous contrast agents.^116^ Exogenous agents can be prepared as required, with an increase of two to three orders of magnitude in molar absorption coefficients in specific wavelengths compared to endogenous contrast agents, which is of benefit to both dynamic imaging and kinetic imaging.^103^ For example, IR‐780 can be used to construct super‐high‐resolution cerebral vascular network structures for hemodynamic studies^103^; 2‐NBDG can be used for quantitative imaging of brain metabolism^10^; hyaluronate–silica nanoparticle can be used as biocompatible liver‐targeted PA contrast agents^153^; indocyanine green can mark lung tumor cells^154^; and gold nanoparticles can be used in the assistant diagnosis of early kidney damage and contrast imaging of cancer cells.^155^ With stronger interdisciplinary collaborations between PACT and materials science researchers, more and more exogenous contrast agents for PACT will be developed in the future for target‐specific functional and molecular imaging.

Since the advent of PACT, many photoacoustic imaging techniques have been developed to improve resolution and penetration depth, such as multispectral technique, multi‐angle illumination technique, and so forth.^28^, ^37^ Internal illumination is a promising technique for PACT imaging of internal organs and tissues in preclinical and clinical practice.^156^ Existing studies have demonstrated the use of internal illumination to image deep structures of the mouse brain and the beating heart of mice under 37 mm thick chicken breast tissue,^16^ and they have shown the ability of internal illumination to realize PACT imaging to a depth of 10 cm through in vitro experiments and in vivo experiments involving renal vascular imaging in live pigs.^42^ The combination of simultaneous external and internal illumination is expected to enable PACT imaging of the whole human brain, as well as the lungs, lymph nodes, heart, kidneys, cervix, and prostate. Internal illumination can also be used with optical contrast agents for functional and molecular imaging of internal tissues and organs inside the body.^103^ With the development of relevant techniques, internal illumination is expected to play a critical role in clinical monitoring of cancer and diseases in internal organs.^7^

PACT has matured greatly through biomedical research over the last two decades. Currently, PACT is capable of whole‐body imaging of mice and organ imaging in mice, rats, pigs, and other animals.^4^ PACT has also been frequently used in preclinical and clinical human body imaging, such as skin, breast, and extremity imaging.^115^ Continuing efforts have been made to achieve PACT imaging of the human brain. We are confident that PACT will have a wide range of applications in biomedical research and clinical practice in the future.

## AUTHOR CONTRIBUTIONS

**Yanru Gu:** Resources (equal); writing – original draft (lead). **Yuanyuan Sun:** Resources (lead); writing – original draft (equal). **Xiao Wang:** Software (equal); writing – original draft (equal). **Hongyu Li:** Resources (equal); writing – original draft (equal). **Jianfeng Qiu:** Resources (equal); writing – review and editing (equal). **Weizhao Lu:** Resources (equal); supervision (lead); writing – review and editing (lead).

## CONFLICT OF INTEREST

The authors declare no potential conflicts of interest.

## PERMISSIONS TO REUSE

All figures are adapted with permission from the publishers or the corresponding authors.

### PEER REVIEW

The peer review history for this article is available at https://publons.com/publon/10.1002/btm2.10419.

## Data Availability

Data sharing is not applicable to this article as no new data were created or analyzed in this study.
